# 

**Published:** 1857-12

**Authors:** 


### DECEMBER CONTENTS.

Girls of New York....... 269
How to Help the Poor....... 270
Rancid Butter, Cured....... 271
"Hubbing"....... 272
Care of the Eyes....... 273
Tooth Powders....... 274
Cup of Tea....... 275
Hot-Air Furnaces....... 276
Warts....... 277
Philosophy of Hunger....... 277
Use of our Exchanges....... 278
Second Childhood....... 281
Healthy Country....... 282
The Best Inheritance....... 282
Child Murder....... 283
Three Articles....... 283
Cheapest Food....... 283
Coffee and Tobacco....... 284
Vermin Riddance....... 285
Intussusception....... 285
Life-Times....... 286
Healthy Recreations....... 286
Patent Medicines....... 287
To Purify Water....... 287
Cure of Corns....... 288
A Patent Dip....... 289
Hearing Improved....... 290
Miscellaneous....... 291
Special Notice....... 291

